# Enhanced reactivity to pain in patients with rheumatoid arthritis

**DOI:** 10.1186/ar2684

**Published:** 2009-05-04

**Authors:** Robert R Edwards, Ajay D Wasan, Clifton O Bingham, Joan Bathon, Jennifer A Haythornthwaite, Michael T Smith, Gayle G Page

**Affiliations:** 1 Department of Anesthesiology, Harvard Medical School, Brigham & Women's Hospital, 850 Boylston Street, Suite 302, Chestnut Hill, MA 02467, USA; 2 Department of Psychiatry, Johns Hopkins University School of Medicine, 600 N. Wolfe Street, Baltimore, MD 21287, USA; 3 Division of Rheumatology, Johns Hopkins University School of Medicine, 5200 Eastern Avenue, MFL Suite 4100, Baltimore, MD 21224, USA; 4 Johns Hopkins University School of Nursing, 525 N. Wolfe Street, Baltimore, MD 21287, USA

## Abstract

**Introduction:**

Maladaptive physiological responses to stress appear to play a role in chronic inflammatory diseases such as rheumatoid arthritis (RA). However, relatively little stress research in RA patients has involved the study of pain, the most commonly reported and most impairing stressor in RA. In the present study, we compared psychophysical and physiological responses to standardized noxious stimulation in 19 RA patients and 21 healthy controls.

**Methods:**

Participants underwent a single psychophysical testing session in which responses to a variety of painful stimuli were recorded, and blood samples were taken at multiple time points to evaluate the reactivity of cortisol, interleukin-6 (IL-6), and tumor necrosis factor-alpha (TNF-α) to the experience of acute pain.

**Results:**

The findings suggest that RA patients display a fairly general hyperalgesia to mechanical and thermal stimuli across several body sites. In addition, while serum cortisol levels did not differ at baseline or following pain testing in patients relative to controls, the RA patients tended to show elevations in serum IL-6 and demonstrated enhanced pain-reactivity of serum levels of TNF-α compared with the healthy controls (*P *< 0.05).

**Conclusions:**

These findings highlight the importance of pain as a stressor in RA patients and add to a small body of literature documenting amplified responses to pain in RA. Future studies of the pathophysiology of RA would benefit from the consideration of acute pain levels when comparing RA patients with other groups, and future trials of analgesic interventions in RA patients may benefit from evaluating the effects of such interventions on inflammatory activity.

## Introduction

Multiple lines of investigation suggest that stress plays a significant role in shaping the course of inflammatory diseases such as rheumatoid arthritis (RA) [1-3]. Stress activates a cascade of neurohumoral events, many of which may be dysregulated in RA patients, including aspects of the hypothalamic-pituitary-adrenal (HPA) axis, the autonomic nervous system, and pro-inflammatory processes [1,3]. Dozens of studies over the past several decades have evaluated the effect of multiple types of stressors on the physiology and symptomatology of patients with RA. Collectively, laboratory research has documented a maladaptively pro-inflammatory response to stress among RA patients, with elevated stress-reactivity of factors such as C-reactive protein (CRP) [4] and tumor necrosis factor-alpha (TNF-α) [5]. Moreover, a relative hypo-responsiveness of the autonomic nervous system and HPA system have been observed in RA patients in response to mental stress as well as a variety of physical stressors [1,3].

Much stress research in RA has been conducted outside of the laboratory, and studies of naturally occurring stressors have revealed that elevations of daily stress among RA patients are associated with increases in musculoskeletal tenderness, interleukin-6 (IL-6) levels, and disease activity [6-9]. Interestingly, relatively little of this research has involved the study of pain, the most commonly reported and most impairing stressor in RA [10]. The experience of pain is generally associated with enhanced release of pro-inflammatory cytokines, which in turn sensitize the nervous system, promoting a further amplification of pain transmission [11-14]. To date, a handful of human studies have documented the presence of cytokine reactivity to the application of calibrated noxious stimuli in humans. Significant increases in pro-inflammatory cytokines such as IL-6 have been observed following non-tissue-damaging painful stimulation in healthy adults [15,16], patients with juvenile RA [17], and patients with persisting low back pain [18].

Given that RA patients experience persistent pain and chronic inflammation, it is natural to inquire whether the inflammatory response to the experience of pain itself is normal in RA. Importantly, psychophysical studies indicate that, relative to controls, RA patients exhibit lower pressure pain thresholds (PPThs) and enhanced sensitivity to noxious stimuli across a variety of anatomical sites, including both inflamed joints and non-inflamed tissues [19-26], suggesting central amplification of pain-related information. This enhancement of pain sensitivity appears to be magnified in individuals with RA of longer duration [25].

To date, although it is well established that RA patients are more behaviorally responsive to noxious stimulation relative to non-arthritic controls, no studies have evaluated whether RA patients show aberrant inflammation-related responses to the experience of acute pain in a controlled laboratory setting. It is important to evaluate the inflammatory response to noxious stimulation among RA patients as daily pain is among their most common and salient stressors. In the present project, we focus on assessing IL-6, TNF-α, and cortisol reactivity to acute painful stimulation in a sample of RA patients compared with age- and gender-matched healthy controls.

## Materials and methods

### Participants

Participants were 19 treated RA patients and 21 generally healthy controls, free from rheumatic disease. RA patients were recruited via letters and flyers sent to patients of the Johns Hopkins Arthritis Center, who were diagnosed with RA using the American College of Rheumatology criteria [27]; controls were recruited through the posting of flyers and the use of newspaper advertisements around the Baltimore community. All subjects provided informed consent, and the study was approved by the Johns Hopkins Institutional Review Board. None of the authors has any financial or other conflicts of interest with regard to this study or its findings.

Inclusion criteria for the study (for RA patients) included RA as the primary source of persistent pain; no current mood or anxiety disorder; no history of myocardial infarction or cardiovascular disease; no history of peripheral neuropathy, Raynaud syndrome, vasculitis, or peripheral vascular disease; no current infection; no history of other autoimmune or rheumatic disorders; and no recent history of substance abuse or dependence. Subjects taking opioid, antidepressant, or steroid medications were not included in the study. Pregnant women were also not included in the study. Healthy controls met all of the same criteria; in addition, they did not have RA or other joint pain and were not taking any centrally acting medications. RA patients reported being on stable treatment regimens for at least 1 month; those taking non-steroidal anti-inflammatory medications were asked to abstain from using them for 24 hours prior to the laboratory session.

### Session protocol

All subjects provided verbal and written informed consent, and all procedures were approved by an institutional review board. Many of these procedures have been described previously [16]. The setting for the study was a general clinical research center (GCRC) based within a university hospital. Participants arrived between 12 and 12:30 p.m.; they had previously been requested to refrain from using over-the-counter medications or caffeine, smoking, or performing other than mild exercise prior to their arrival. To avoid interfering with RA treatment regimens, participants were asked to take their RA medications as prescribed. After informed consent and screening for eligibility, participants completed questionnaires for approximately 10 minutes. Questionnaires included a medical history form, questions about current pain and current stress levels (rated on 0-to-10 scales), the Beck Depression Inventory (BDI) [28], and the Short Form Health Survey-36 (SF-36) [29]. Determination of eligibility for the study was made based on questionnaires and a medical history taken by a research nurse at the GCRC.

Next, subjects were seated comfortably in a reclining chair and an intravenous (i.v.) line was inserted in the left forearm by a GCRC research nurse [17,30]. After i.v. placement and a 15-minute period of rest, two baseline blood samples (10 mL), separated by 5 minutes, were drawn. These two values were averaged together in order to maximize stability of the baseline estimates. Baseline systolic and diastolic blood pressures were then recorded. Subsequently, participants underwent the psychophysical pain testing procedures described below (the duration of pain testing was approximately 45 minutes), after which additional blood samples (10 mL) were taken at several time points: immediately after testing and 15, 30, and 60 minutes after testing.

### Psychophysical pain testing (45-minute session)

Mechanical pain thresholds were assessed first using a digital pressure algometer (Somedic Production AB, Sollentuna, Sweden). As in previous studies [19,21,23], we selected several muscle/joint sites and bilaterally assessed PPThs. PPThs were determined twice at each of the following sites on the right and left sides of the body in a randomized order: the belly of the trapezius muscle, the metacarpophalangeal joint of the thumb, and the quadriceps muscle, near the insertion of the proximal patellar tendon. At each site, mechanical force was applied using a 0.5-cm^2 ^probe covered with polypropylene pressure-transducing material; pressure was increased at a steady rate of 30 kPa/second until the subject indicated that the pressure was 'first perceived as painful'.

Next, contact heat stimuli were delivered using a Medoc Thermal Sensory Analyzer (TSA-2001; Medoc Ltd., Ramat Yishai, Israel). Thermal assessment included sampling of heat pain thresholds (HPThs) on the ventral forearm using an ascending method of limits paradigm with a rate of rise of 0.5°C/second [31]. Three trials of HPTh were performed first, followed by four trials of suprathreshold heat stimulation. In brief, four sequences of 10 rapid heat pulses were applied to the forearm, similar to prior studies [32,33]. Within each sequence, the procedure was as follows: from a 38°C baseline temperature, 10 successive thermal pulses were delivered. The rate of rise and fall of the thermode temperature was 10°C/second, and target temperatures were delivered for approximately 0.5 seconds each. The thermode remained in a fixed position during administration of the 10 pulses and then was re-positioned between sequences, with inter-sequence intervals of 2 minutes. Two different target temperatures (49°C and 51°C) were used two times each in randomized order. Subjects verbally rated the painfulness of each thermal pulse on a 0-to-100 (0 = 'no pain' and 100 = 'most intense pain imaginable') numeric rating scale and then rated the painfulness of lingering after-sensations 15 seconds after the stimuli had ceased [34,35].

Finally, responses to noxious cold were evaluated using a repeated cold pressor task (CPT), involving immersion of the right hand in a circulating cold water bath maintained at 4°C. The CPT is the most commonly used method of pain induction in the laboratory and has demonstrated clinical relevance [36,37]. Several recent studies indicate that the CPT provokes increases in cortisol and norepinepherine as well as producing increases in pro-inflammatory cytokine production [16,17]. In the present protocol, participants underwent a series of five CPTs, with the first four consisting of serial immersions of the right hand for 30 seconds, with 2 minutes between immersions. The fifth and final CPT involved an immersion of the right hand lasting until a participant reached pain tolerance (or a 3-minute maximum). Participants rated the intensity of the cold pain on a 0-to-100 scale ('no pain' to 'most intense pain imaginable') at the midpoint and conclusion of each CPT. Following the final CPT, participants continued to relax in the chair as subsequent blood samples were taken.

### Physiological measures

Each blood sample (that is, two baseline samples, one sample immediately after pain testing, then samples at 15, 30, and 60 minutes following the conclusion of pain testing) was collected in a 10-mL tube and transported to the GCRC Core Laboratory, where it was centrifuged, aliquoted, and stored in a -80°C freezer for later assay. Serum cortisol was assessed in duplicate using a radioimmunoassay (Diagnostic Systems Laboratories, Inc., Webster, TX, USA), with a lower limit of detection of 0.5 μg/dL, a sensitivity of 0.11 μg/dL, and an intra-assay coefficient of variation of less than 10%. A standard high-sensitivity enzyme-linked immunosorbent assay (R&D Systems, Minneapolis, MN, USA) was used to assess serum levels of IL-6 in duplicate. This assay has a lower limit of detection of 0.16 pg/mL, a sensitivity of 0.04 pg/mL, and an intra-assay coefficient of variation of less than 5%. Similarly, an enzyme-linked immunosorbent assay from the same company (R&D Systems) was used to assess serum levels of TNF-α in duplicate. This assay has a lower limit of detection of 0.25 pg/mL, a sensitivity of 0.06 pg/mL, and an intra-assay coefficient of variation of less than 10%.

### Data analysis

Simple between-group comparisons (RA patients compared with controls) were made using analysis of variance (ANOVA). Changes, across the two groups, in serum levels of cortisol, IL-6, and TNF-α were evaluated using repeated measures ANOVA. Inter-relationships among study variables were evaluated using Pearson correlations. All analyses were performed using SPSS (SPSS Inc., Chicago, IL, USA).

## Results

RA patients reported a mean time since diagnosis of 8.3 years (standard deviation = 6.4 years). The mean disease activity score using 28 joint counts (DAS28) for the sample was 3.1 ± 1.4. In addition, the mean CRP level in RA patients was 3.3 ± 3.9 μg/ml. These values suggest generally low to moderate levels of disease activity, on average, in these patients and are broadly consistent with other, larger US studies of treated RA patients (for example, in [38], mean RA duration = 12.4 years, mean DAS28 score = 3.7, and median CRP = 2.6 μg/ml).

RA patients did not differ (all *P *values of greater than 0.10) from controls on demographic variables such as age (mean age for RA patients = 51.7 ± 12.2 years and mean age for controls = 50.3 ± 12.7 years), gender (58% women in the RA group and 52% women in the control group), ethnicity (58% in the RA group were white and 67% in the control group were white), or education (mean years of education for RA patients = 14.0 ± 2.7 and mean years of education for controls = 15.1 ± 2.5). In addition, CRP levels in RA patients (mean = 3.3 ± 3.9) did not differ significantly from CRP levels in controls (mean = 2.5 ± 3.5). Finally, resting systolic blood pressures (SBPs) in the controls (mean = 122.8 ± 9.6 mmHg) did not differ from SBPs in the RA patients (mean = 122.1 ± 18.8 mmHg). Similarly, diastolic blood pressures in the controls (mean = 70.1 ± 6.0 mmHg) and RA patients (mean = 64.4 ± 10.7 mmHg) were similar (*P *> 0.10).

All RA patients were receiving treatment for their disease, though with significant variability in the treatment regimens. The following is a summary of the disease-modifying antirheumatic drugs (DMARDs) taken by the 19 RA patients in this study: methotrexate (MTX) monotherapy (n = 8), hydroxychlorochloroquine monotherapy (n = 2), TNF antagonist monotherapy (n = 3), MTX + other non-biologic DMARD (n = 4), and MTX + TNF antagonist (n = 2).

### Questionnaires

In terms of questionnaire responses, RA patients did report higher levels of current and recent pain and lower scores on indices of health and physical functioning relative to the controls (Table 1). Interestingly, patients and controls did not differ on self-report of current stress levels or the SF-36 indices of mental/emotional health. RA patients did endorse higher scores on the BDI, although mean levels of depressive symptoms were low and within the normal range (that is, BDI scores of less than 10 are generally considered subclinical) for both groups.

**Table 1:** Comparison of rheumatoid arthritis patients and controls on pain and questionnaire responses

	RA patients (n = 19)	Controls (n = 21)	*P *value
Responses to noxious stimuli			
HPTh, °C	41.4 ± 5.1	44.4 ± 4.5	0.05
PPTh on leg, kPa	665.5 ± 287.7	811.3 ± 400.1	0.19
PPTh on thumb, kPa	295.7 ± 141.6	395.7 ± 150.7	0.03
PPTh on trapezius, kPa	404.5 ± 160.7	536.2 ± 276.9	0.08
Cold pain rating (0 to 100) at midpoint	82.3 ± 12.6	65.4 ± 25.6	0.01
Cold pain rating (0 to 100) at conclusion	83.0 ± 12.4	67.7 ± 24.8	0.02
Cold pain tolerance, seconds	61.8 ± 54.1	111.8 ± 63.8	0.01
Heat pain rating at 49°C	74.4 ± 25.1	57.7 ± 34.7	0.09
Heat pain rating at 51°C	86.8 ± 16.2	68.2 ± 32.4	0.03
Painful heat after-sensations	16.8 ± 23.2	5.7 ± 9.4	0.05
Questionnaire data			
Current pain (0 to 10)	3.2 ± 2.3	0.4 ± 0.3	< 0.001
Current stress (0 to 10)	2.2 ± 2.4	1.2 ± 1.9	0.17
Beck Depression Inventory score	7.0 ± 6.3	2.5 ± 3.0	0.01
SF-36, subscale score			
General health	52.9 ± 20.3	84.0 ± 19.4	< 0.001
Physical functioning	42.1 ± 24.2	69.1 ± 26.7	0.002
Physical role	28.9 ± 31.5	86.9 ± 28.1	< 0.001
Bodily pain	44.2 ± 21.3	85.7 ± 23.7	< 0.001
Energy/fatigue	53.4 ± 18.4	67.6 ± 18.1	0.02
Mental health	80.7 ± 13.7	81.0 ± 14.5	0.95
Emotional role	84.2 ± 34.0	93.7 ± 22.7	0.30
Social functioning	70.4 ± 27.7	94.6 ± 7.5	0.001

Data are presented as mean ± standard deviation. HPTh, heat pain threshold; PPTh, pressure pain threshold; RA, rheumatoid arthritis; SF-36, Short Form Health Survey-36.

### Pain responses

Comparisons between RA patients and controls on measures of psychophysical pain responses yielded statistically significant (*P *≤ 0.05) or near-significant differences on a number of measures. RA patients had lower HPThs, lower mechanical pain thresholds on the thumb, higher pain intensity ratings of 51°C heat stimuli and heat after-sensations, lower cold pain tolerance, and higher cold pain ratings during the CPT tests. Tendencies that did not reach the level of frank statistical significance were noted for PPTh on the trapezius and heat pain ratings in response to the 49°C stimuli. These data are presented in Table 1.

### Physiological responses

**Figure 1:**
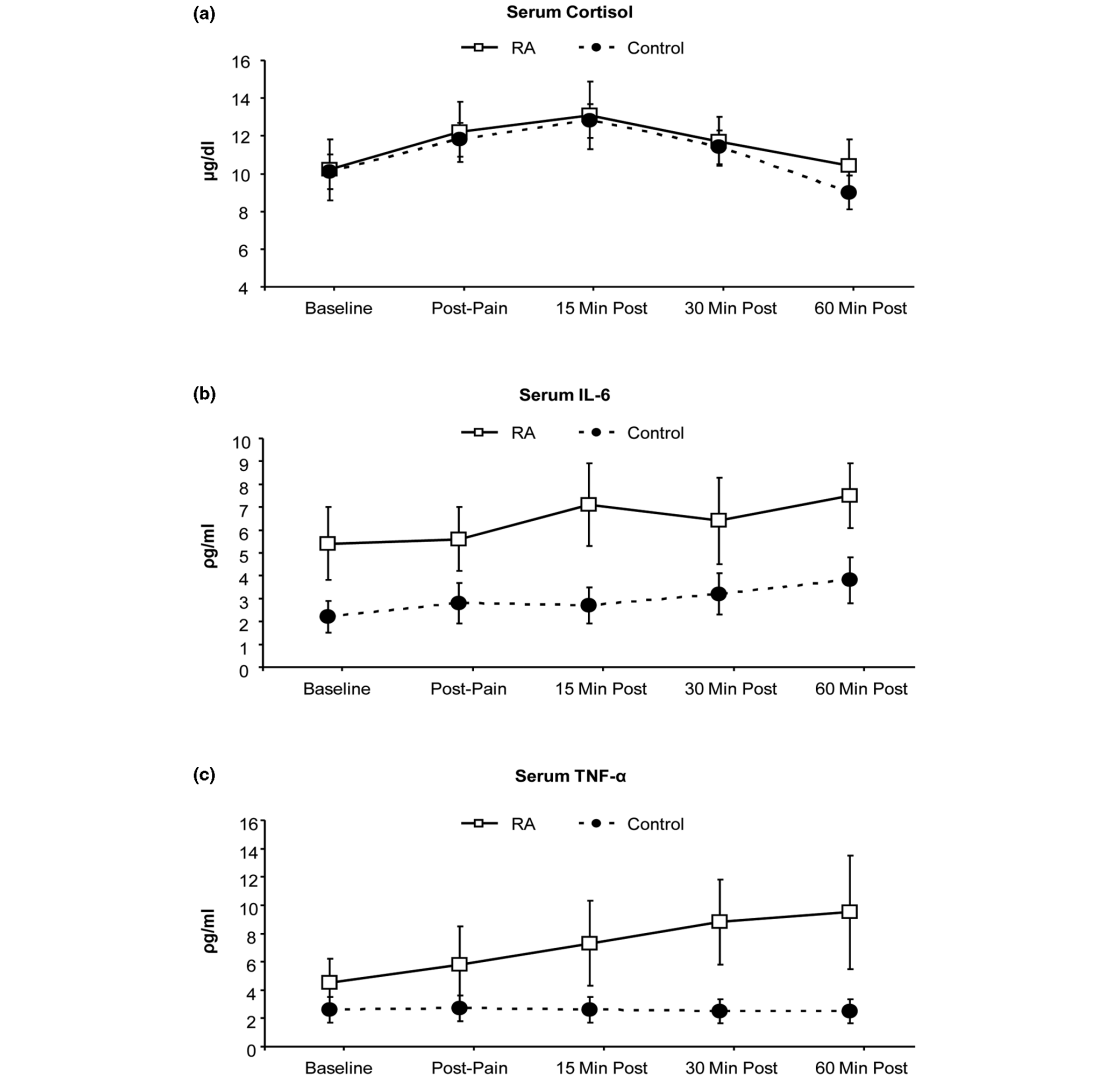
Changes in serum levels of **(a)** cortisol,** (b)** interleukin-6 (IL-6), and **(c)** tumor necrosis factor-alpha (TNF-α) over the course of the session. Data are presented as mean ± 95% confidence interval. RA, rheumatoid arthritis.

Repeated measures ANOVAs were used to evaluate between-group differences in levels of cortisol, IL-6, and TNF-α over the course of the session. As the demographics of the groups were similar, we did not control for age, gender, race, or education, but SF-36 general health subscale scores were entered as a covariate in order to statistically control for clear group differences in perceived health. For measures of serum cortisol, there was a strong main effect of time [F(4,34) = 8.3, *P *< 0.01], but no significant main effect of group or group × time interaction (*P *> 0.1). For IL-6, there was also a main effect of time [F(4,34) = 4.0, *P *< 0.01] as well as a trend for a main effect of group [(F(1,37) = 3.2, *P *= 0.07]. On average, the RA patients had serum IL-6 levels that tended to be higher than those of the controls at every time point. The IL-6 data showed no interaction between group × time. Finally, for the TNF-α data, the main effects of time and group were qualified by a significant interaction [F(4,34) = 3.3, *P *= 0.02]. Among the RA patients, serum TNF-α increased significantly from baseline following the pain testing (*P *< 0.05), whereas no significant changes in TNF-α were observed in the controls. Cortisol, IL-6, and TNF-α data are depicted in Figure 1.

Although our sample of 19 RA patients is too small to permit extensive investigation of the relationships between cytokine responses to pain and clinical variables, we assessed correlations of TNF-α and IL-6 responses with the SF-36 subscales of bodily pain, energy/fatigue, and physical functioning. Within the RA group, TNF-α levels were unrelated to bodily pain or physical functioning but showed a tendency to relate to lower levels of energy (or higher levels of fatigue): *r *= -0.43, *P *= 0.07. IL-6 levels were similarly associated with bodily pain (*r *= -0.41, *P *= 0.08), energy/fatigue (*r *= -0.45, *P *= 0.06), and physical functioning (*r *= -0.42, *P *= 0.08).

## Discussion

The present findings are consistent with previous research suggesting that RA patients exhibit reduced quality of life relative to controls [39-41]. Interestingly, though, these effects are relatively specific in the present study to measures of pain and physical functioning (that is, the RA and control groups did not differ on the SF-36 subscales that evaluate mental health and emotional functioning). Moreover, our findings complement previous work indicating that individuals with RA are more sensitive to a variety of modalities of noxious stimulation relative to a healthy comparison group [19-26]. These data suggest that RA patients display hyperalgesia to mechanical and thermal stimuli at both disease-affected sites (that is, PPTh on the thumb was lower in RA patients relative to controls) and many non-joint sites (that is, on the skin of the forearm, HPThs were lower and heat pain ratings were higher in RA patients). The generalized nature of the enhanced sensitivity to pain observed in these patients suggests alterations in pain processing at the level of the central nervous system, as we [42] and others [43,44] have hypothesized.

To our knowledge, this is the first investigation to report differences between RA patients and controls in physiological responses to acute, standardized, non-tissue-damaging, noxious stimulation. Although prior work had indicated that stress is likely to play a significant role in the maladaptive functioning of neuroendocrine and inflammatory processes in patients with RA [1-3], the physiological perturbations associated with pain perception had not previously been evaluated. The present findings reveal that, in treated RA patients compared with controls, acute pain induction is associated with elevations in serum TNF-α levels that last for at least 1 hour. These data are consistent with the notion that the experience of pain is associated with enhanced release of pro-inflammatory cytokines, which in turn sensitize the nervous system, promoting a further amplification of pain transmission [11-14]. While several other human studies had documented the presence of cytokine reactivity to the application of calibrated noxious stimuli [15,16,18], these results indicate that such reactivity (at least for TNF-α) may be magnified in the context of RA. Stressors such as pain activate a cascade of neurohumoral events, many of which may be dysregulated in RA patients, who show a maladaptively pro-inflammatory response to various types of stress [4,5]. Moreover, a relative hypo-responsiveness of the autonomic nervous system and HPA system have been observed in RA patients [1,3,45,46], although we did not find group differences in this study in the response of cortisol to acute pain. The acute increase in cortisol following painful stimulation is consistent with prior studies [47], but it is important to note that stress responses in RA patients are complex and vary as a function of the stimulus. For example, in contrast to pain as a stressor, exercise stress does not induce cortisol increases in either RA patients or controls [48]. However, an insulin tolerance stress test resulted in a finding of hypocortisolemia among the RA patients relative to controls [49], and similar results were obtained using a combined stressor of exercise, cold pain, and mental stress [50]. Thus, rather than a global generalized hypo-responsiveness of the HPA axis to stress in RA, there appears to be a significant stimulus specificity to stress response profiles.

The greater reactivity of TNF-α and the potentially chronic elevations in IL-6 levels in RA patients are likely to have deleterious long-term consequences. TNF-α upregulates a number of inflammatory processes, and the resulting inflammatory cascade leads directly to joint-damaging events such as cartilage breakdown and resorption of bone. In addition, IL-6 induces muscle and joint hyperalgesia [51,52] and mediates the development of injury-induced hyperalgesia [53]. Following surgery, IL-6 levels are associated with postoperative pain [54-56] and reduced functioning [57]. Even in this small sample of RA patients, we find suggestive correlations of TNF-α and IL-6 levels with indices of fatigue, pain, and physical function. In the future, longitudinal studies will likely be helpful in evaluating potential causal links between cytokine reactivity to acute pain and outcomes such as physical disability and joint damage. In addition, larger-sample studies that can group RA patients as a function of treatment (for example, using TNF antagonists versus not) will be important in evaluating the role of differing pharmacologic regimens in shaping these associations. It is especially interesting that the present findings were observed in a sample of treated RA patients with, on average, low to moderate levels of disease activity and CRP levels that were not different from the controls.

Some important limitations of this study will need to be addressed in later research. We did not include a pain-free control session and hence we cannot exclude the possibility that the elevated TNF-α reactivity in the RA patients was due to factors other than pain. In addition, our measure of TNF-α reactivity showed no sign of decline at our final assessment point, 1 hour after the end of painful stimulation. Thus, we are not able to determine the full time course of this reactivity to pain and it is possible that the increases in TNF-α in the RA patients continued over longer durations. It would also have been desirable to obtain measurements, at the same time points, on other factors that have been linked to pain responses such as anti-inflammatory cytokines [58], catecholamines [59], growth hormone [60], and blood pressure reactivity (a useful index of sympathetic nervous system activation in the context of pain responses [61,62]). Also lacking in this study were any data on prior food consumption during the day of testing. Although we standardized the time of day, the timing and content of a meal can influence basal cytokine levels [63,64]. Future studies in this area may wish to more stringently control for such factors. Finally, this cross-sectional study does not have the capacity to determine the causal links between RA disease processes and cytokine reactivity to pain. It is possible, for example, that pre-existing individual differences in pro-inflammatory cytokine responses to acute stress, perhaps conferred by genotype or early environmental experience, represent a risk factor for the development of RA or other systemic inflammatory diseases. Alternatively, dysregulation of stress responses may be solely a function of the disease itself. Additional longitudinal research methodologies will be necessary to illuminate such questions.

In spite of these limitations, this study highlights the importance of pain and stress in patients with RA. It is important to note that a handful of studies have suggested that, under non-stress conditions, basal TNF-α levels may be comparable between RA patients and controls [65,66]. In the present investigation, we find that, at baseline, serum TNF-α does not differ significantly between groups; it is only following the stressor of acute pain that differences between RA patients and controls emerge. Future studies of the pathophysiology of RA would likely benefit from the consideration of such acute stress and pain levels. Moreover, future clinical trials of analgesics in RA may provide opportunities to examine the effects of pain-relieving treatment on inflammatory activity. Finally, in future studies, the isolation of specific cell populations in cytokine assays or the use of stimulation techniques that permit quantification of cytokine production on a 'per-cell' basis [5] would potentially provide valuable information about the molecular and cellular processes that underpin these observed findings.

## Conclusions

Compared with controls, RA patients show elevations in pain sensitivity in response to multiple stimulus modalities across several body sites. In addition, RA patients display higher levels of serum IL-6 and enhanced pain-reactivity of serum levels of TNF-α. Abnormal pro-inflammatory responses to painful stimulation may play a deleterious role in shaping the long-term symptomatology of RA.

## Abbreviations

ANOVA: analysis of variance; BDI: Beck Depression Inventory; CPT: cold pressor task; CRP: C-reactive protein; DAS28: disease activity score using 28 joint counts; DMARD: disease-modifying antirheumatic drug; GCRC: general clinical research center; HPA: hypothalamic-pituitary-adrenal; HPTh: heat pain threshold; IL-6: interleukin-6; i.v.: intravenous; MTX: methotrexate; PPTh: pressure pain threshold; RA: rheumatoid arthritis; SBP: systolic blood pressure; SF-36: Short Form Health Survey-36; TNF: tumor necrosis factor.

## Competing interests

The authors declare that they have no competing interests.

## Authors' contributions

RRE conceived of the study, analyzed the data, and drafted the manuscript. ADW assisted with interpretation of results and drafting of the manuscript. COB and JB participated in the design and coordination of the study, assisted with patient recruitment, and helped to draft the manuscript. JAH and MTS participated in the conception and design of the study, oversaw data collection, and assisted with data analysis and interpretation. GGP assisted with conduct, analysis, and interpretation of the assays. All authors read and approved the final manuscript.
